# Interaction of Uric Acid and Neutrophil-to-Lymphocyte Ratio for Cardiometabolic Risk Stratification and Prognosis in Coronary Artery Disease Patients

**DOI:** 10.3390/antiox11112163

**Published:** 2022-10-31

**Authors:** Serena Del Turco, Luca Bastiani, Fabrizio Minichilli, Patrizia Landi, Giuseppina Basta, Alessandro Pingitore, Cristina Vassalle

**Affiliations:** 1 Institute of Clinical Physiology, National Research Council, 56124 Pisa, Italy; 2 Fondazione CNR-Regione Toscana Gabriele Monasterio, 56124 Pisa, Italy

**Keywords:** uric acid, neutrophil-to-lymphocyte ratio, cardiometabolic disease, cardiac mortality, hard events

## Abstract

Oxidative stress and inflammation are key factors in cardiometabolic diseases. We set out to evaluate the relationship between serum uric acid (UA) and the neutrophil-to-lymphocyte ratio (NLR) with cardiometabolic risk factors in coronary artery disease (CAD) patients, and their additive and multiplicative interactive effects on outcomes (cardiac death/CD and hard events (HE)—death plus reinfarction). A total of 2712 patients (67 ± 11 years, 1960 males) who underwent coronary angiography was retrospectively analyzed and categorized into no-CAD patients (*n* = 806), stable-CAD patients (*n* = 1545), and patients with acute myocardial infarction (AMI) (*n* = 361). UA and NLR were reciprocally correlated and associated with cardiometabolic risk factors. During a mean follow-up period of 27 ± 20 months, 99 ± 3.6% deaths, and 213 ± 7.8% HE were registered. The Kaplan–Meier survival estimates showed significantly worse outcomes in patients with elevated UA or NLR levels. Multivariate Cox regression analysis demonstrated that NLR independently predicted CD and HE. There was no multiplicative interaction between UA and NLR; however, the use of measures of additive interaction evidenced a positive additive interaction between UA and NLR for CD and HE. Although it is clear that correlation does not imply causation, the coexistence of NRL and UA appears to have a synergistic effect, providing further information for the risk stratification of CAD patients.

## 1. Introduction

Oxidative stress and inflammation are common features of cardiovascular and metabolic diseases [1]. Uric acid (UA), a biomarker easily and reliably measured in clinical practice, is a powerful scavenger of free radicals, although it may also act as a pro-oxidant effector, depending on the surrounding microenvironment [2]. Even though UA is an efficacious oxygen scavenger and its antioxidant effects prevent metabolic alterations, the occurrence of hyperuricemia may represent a maladaptive response put in place to address excessive oxidative stress, which may contribute to the onset and development of cardiometabolic disease [2]. Principal UA-related mechanisms contributing to these adverse effects involve the induction of systemic inflammation and the modulation of several pathways associated with oxidative stress, insulin resistance/diabetes, and endothelial dysfunction [3]. Accordingly, serum uric acid has been found associated with coronary atherosclerosis, the severity of coronary artery lesions, and cardiovascular and all-cause mortality [4].

Lymphocytes and neutrophils play a central role in the inflammatory response. The neutrophil-to-lymphocyte ratio (NLR), a simple hemochrome-derived index practically always available as part of the general patient evaluation, is an index of systemic inflammation and immunity and is emerging as a prognostic biomarker in many diseases, including the cardiovascular diseases [5]. NLR seems a more efficacious predictor than the total white blood count or any of the leukocyte subtypes. In fact, this parameter represents a ratio of two opposite but complementary immune pathways, reflecting the effect of the neutrophils (nonspecific immune response), as well as lymphocytes (specific immune response). 

Although an association between hyperuricemia and NLR has been observed in cardiometabolic diseases, the combined effect of NLR and uric acid increase on cardiometabolic components and prognosis in stable coronary artery disease (CAD) is inadequately defined [6,7]. Thus, in this study, we aimed to evaluate whether the combined elevation of NLR and UA may have additive values with respect to the increase of each biomarker on the cardiometabolic risk stratification and outcomes in patients with stable CAD.

## 2. Materials and Methods

### 2.1. Patients

A total of 2712 patients (mean age (SD): 67 (11) years, 1960 males) who underwent coronary angiography at the Cardiology Department of the Institute of Clinical Physiology–CNR (Pisa, Italy) was retrospectively analyzed by using the Institute’s electronic databank (installed in 1975), collecting detailed information about the demographic, clinical, laboratory, and instrumental data of consecutive patients hospitalized from 1977 to 2011 [8]. Patients were categorized into three groups based on their clinical presentation and coronary angiography results: (1) no-CAD group, including 806 patients with no stenosis in any coronary artery; (2) stable CAD group, including 1545 stable CAD, confirmed by coronary angiography, with a ≥50% diameter stenosis in at least one major coronary artery; (3) AMI group made of 361 patients with acute myocardial infarction (acute CAD) [9].

The inclusion criterion in the study was the absence of history or clinical or instrumental evidence of atherosclerosis in vascular districts other than the coronary bed. Exclusion criteria were any predominant non-cardiac chronic diseases (e.g., infection, acute or chronic inflammatory disease, severe valvular heart disease, renal or hepatic insufficiency, autoimmune disease) or known malignant diseases. Patients taking UA-lowering medications (e.g., allopurinol) were also excluded. A complete clinical history of each subject was available, including cardiovascular risk factors such as smoking history (current or past-smoking habit), hypertension (blood pressure higher than 140/90 mmHg or the use of antihypertensive drugs), diabetes (T2D: fasting glycemia >126 mg/dl or use of an antidiabetic treatment), and dyslipidemia (total cholesterol concentration ≥220 mg/dL, or triglyceride concentration ≥200 mg/dL, or current use of lipid-lowering drugs). Patient medications included nitrates, oral aspirins, calcium antagonists, angiotensin-converting enzyme inhibitors, and diuretics.

Biochemical analysis was performed at patient admission, and blood samples were drawn after an overnight fast and immediately assessed by routine automated laboratory analyzers.

### 2.2. Angiographic Study

All patients underwent coronary angiography. The severity of CAD was determined by the number of involved main coronary arteries (one-, two-, or three-vessel disease) with a luminal diameter narrowing of >50%, and all angiograms were reviewed by expert cardiologists. 

### 2.3. Follow-Up

As previously described, follow-up events were collected retrospectively from at least one of the following four methods: a review of the patient’s record, telephone interview conducted by trained personnel, personal communication with the patient’s physician, or medical visit to the outpatient clinic [10,11]. The clinical events recorded and analyzed for the prediction of events were cardiac death, and total death plus non-fatal myocardial infarction (hard events-HE). The cause of cardiac death was derived from medical records or death certificates and required the documentation of either significant arrhythmias, cardiac arrest, death attributable to congestive heart failure, or myocardial infarction in the absence of any other precipitating factor. The diagnosis of myocardial infarction was based on the documentation of persistent electrocardiographic ST segment changes, or new Q wave development, associated with biomarker increase.

### 2.4. Statistical Analysis

Data are expressed as mean standard deviation (SD). The statistical analyses performed included the Student’s *t*-test, the chi-square test and analysis of variance (ANOVA), and the post hoc Scheffe’s test. Multivariate regression analysis was performed to identify UA- and NLR-independent determinants.

Cumulative event rates were estimated by Kaplan–Meier survival curves, and probability values were determined with the log-rank test. For survival analysis, only one event was considered in each patient. Statistical analysis also included Cox proportional hazard models to determine independent predictors of cardiovascular events (CD and HE).

To assess the biological interaction between UA and NLR, the following parameters were calculated [12]:

#### 2.4.1. Relative Excess Risk (RERI)

RERI was defined as the additional risk due to interaction (part of the total effect that is due to interaction) on an additive scale, and it was calculated as follows:

RERI = HR 11−HR 01−HR 10 + 1, where HR 11 refers to the HR for high UA and high NLR; HR 01 is the OR for low UA and high NLR, and HR 10 represents exposure to high UA and low NLR. The RERI of zero indicates no additive interaction, whereas a RERI greater than 0 indicates an additive interaction.

#### 2.4.2. Attributable Proportion (AP)

The proportion attributable to interaction (proportion of the combined effect that is due to interaction), the AP estimate, indicated the proportion of risk attributable to the interaction of the high UA and high NLR, and it was calculated according to the following formula:

AP = (HR11−1)/((HR1−1) + (HR10−1)), with AP > 0, which indicates biological interaction.

#### 2.4.3. Synergy Index (SI)

SI was calculated as the ratio between the combined effect and individual effects, according to the formula: SI = RERI/HR 11. SI estimates whether a synergistic (SI > 1) or antagonistic (SI < 1) interaction exists between two exposures, where SI = 1 means no interaction.

#### 2.4.4. Multiplicative Interaction Ratio of HRs

The ratio of HRs = HR 11/(HR 10 × HR 01) if equal to one indicates there is no multiplicative interaction.

Statistical analysis was performed using the statistical package Statview, version 5.0.1 (SAS Institute, Abacus Concept, Inc., Berkeley, CA, USA). A *p*-value ≤ 0.05 was considered significant.

## 3. Results

### 3.1. Characteristics of the Study Participants

Demographic and clinical characteristics of patients according to coronary angiography results and clinical presentation (*n* = 806 no-CAD, 1545 CAD, 361 AMI, respectively) are reported in Table 1. The overall population included 2712 patients (1960 males) with a mean age of 67 years, and means of UA and NLR accounted for 6.1 ± 1.6 mg/dL and 3.0 ± 3.3, respectively. As expected, patients with CAD (stable or acute) were older, with a predominance of the male gender, and more frequently presented with cardiometabolic risk factors. NLR was significantly higher in the AMI group, although no difference was observed for UA levels between the three groups.

### 3.2. Determinants of UA

UA levels directly correlated with aging (*r* = 0.1, *p* < 0.001), BMI (*r* = 0.15, *p* < 0.001), and NLR (*r* = 0.1, *p* < 0.001), and inversely with EF (*r* = −0.24, *p* < 0.001). Moreover, UA was higher in males (6.3 ± 1.5 vs. 5.6 ± 1.7 mg/dL in females, *p* < 0.001) in patients presenting hypertension (6.2 ± 1.6 and 6.0 ± 1.6 mg/dL, *p* < 0.05) and T2D (6.4 ± 1.8 and 6.0 ± 1.5 mg/dL, *p* < 0.001) with respect to those without these risk factors. Moreover, UA was higher in patients presenting multivessel disease concerning mono-a vessel disease or no-vessel disease (6.2 ± 1.6 vs. 6.0 ± 1.5 and 6.0 ± 1.7 mg/dL, respectively, *p* < 0.01).

When the number of risk factors was considered (including diabetes, dyslipidemia, hypertension, and obesity), UA levels showed a progressive increase (from 5.9 ± 1.7 in absence of risk factor, to 6 ± 1.5 in presence of one/two risk factors, and 6.4 ± 1.7 for patients presenting two or more risk factors, *p* < 0.001).

Multivariate regression analysis identified male gender, aging, BMI, NLR, T2D, hypertension, and EF as significant determinants of UA levels (Table 2). 

### 3.3. Determinants of NLR

A positive relationship was found between NLR and age (*r* = 0.1, *p* < 0.001), and an inverse correlation was found between NLR and BMI (*r* = −0.1, *p* < 0.001) and EF (*r* = −0.14, *p* < 0.001). A significant difference in NLR levels was observed between patients with and without T2D (3.5 ± 3.8 and 2.8 ± 3.0, *p* < 0.001, respectively), with and without dyslipidemia (2.9 ± 3.2 and 3.3 ± 12, *p* < 0.01, respectively), and with and without hypertension (2.8 ± 2.7 and 3.1 ± 3.9, *p* < 0.05, respectively). Moreover, NLR progressively increased in patients presenting no-vessel disease when compared to those with mono-vessel disease or multivessel disease (2.6 ± 3.4 vs. 2.9 ± 2.9 and 5.3 ± 4.3 mg/dL, respectively, *p* < 0.001). NLR did not change with the number of cardiovascular risk factors (from 3 ± 3.2 in the absence of risk factors, to 3 ± 3.4 in presence of one/two risk factors, and 2.9 ± 2.9 in patients with two or more risk factors).

Multivariate regression analysis identified aging, BMI, UA, T2D, dyslipidemia, multivessel disease, and EF as significant determinants for NLR levels (Table 3).

### 3.4. UA and NLR as Determinants of Outcomes

For logistic analysis, NLR values were stratified according to the 75th percentile corresponding to 3.2, and hyperuricemia was defined as a serum uric acid level >7.0 mg/dL in males and >6.0 mg/dL in females [13,14]. Moreover, age was scored according to the median value (50th percentile: 68 years) and EF as <50%. 

**Figure 1:**
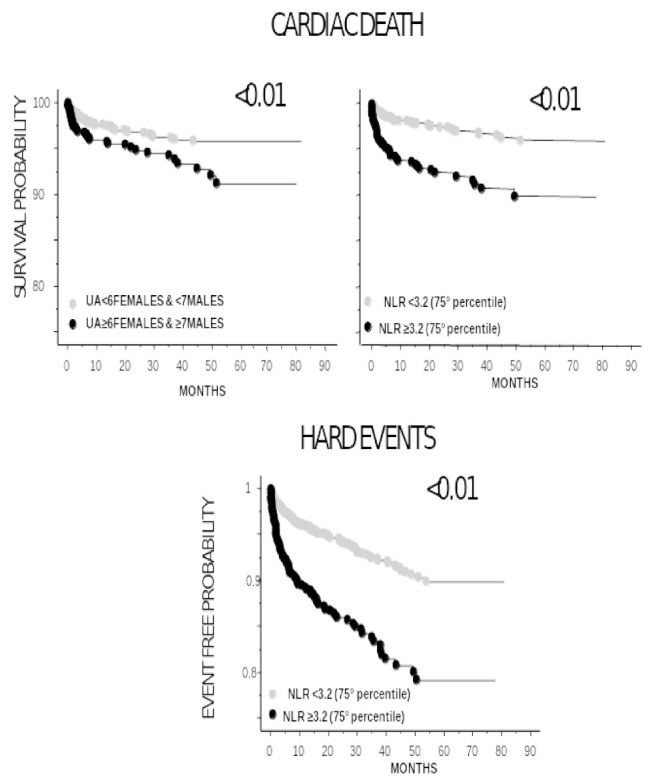
Kaplan–Meier survival curves according to UA and NLR levels for cardiac death and hard events (death plus reinfarction) as endpoints.

During a mean follow-up period of 27 ± 20 months, 99 (3.6%) cardiac death were registered, and 213 (7.8%) had major adverse cardiovascular events (death or reinfarction). The Kaplan–Meier survival estimates showed significantly worse outcomes in patients with elevated UA (cardiac death) or NLR levels (cardiac death and HE) (Figure 1).

The Multivariate Cox regression model identified elevated levels of NLR as an independent determinant associated with a higher risk of death and HE during follow-up (Table 4). 

### 3.5. Interactive Effect of NLR and UA for Cardiac Death and Hard Events

**Figure 2:**
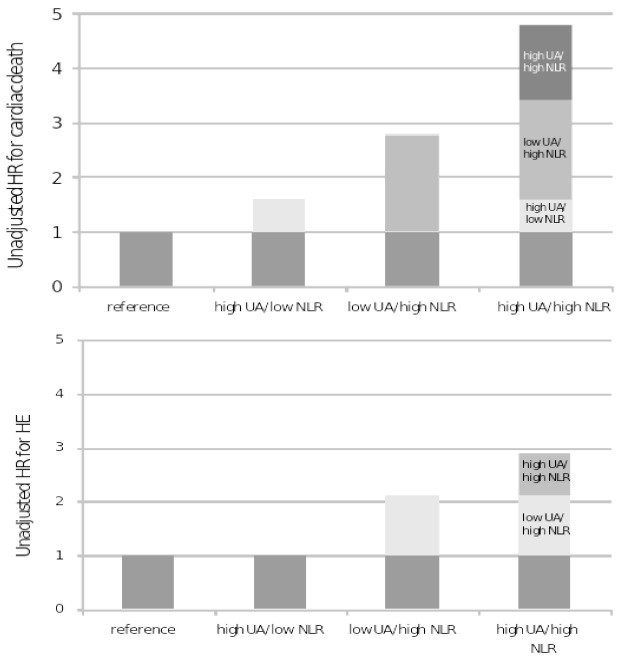
Hazard ratios with contributions from different categories of UA and NLR for cardiac death and hard events.

As shown in Figure 2, in the unadjusted model when compared to the patients with low UA and low NLR (reference), patients with low UA high NLR, and high UA high NLR had an increased risk of cardiac death (unadjusted HR (95% CI), p: 2.8 (1.7–4.7) < 0.001, 4.7 (2.7–8.2) < 0.001, respectively), but not those with high UA low NLR (1.6 (0.9–2.8) < 0.1). RERI > 0, AP > 0, and S > 1 indicated an additive positive interaction. For example, based on unadjusted analysis (see Table 5) there were 1.4 relatively excess risks due to the additive interaction (RERI). Moreover, AP suggested that a total of 30% of the effects (patient cardiac deaths) were estimated to be attributed to the interaction between the two biomarkers. Finally, the synergy index (SI) was 1.6, suggesting that the risk of subjects presenting both risk factors was 1.6 times as high as the sum of risks in the participants exposed to each risk factor alone. These findings mean that when high UA and high NLR coexist, the estimated effect is greater than the sum of high UA and high NLR separately. However, there was no evidence of interaction on the multiplicative scale.

For HE, although the contribution of high UA/low NLR was negligible, a weak positive interaction was appreciable after the combination of UA with high NLR (Table 6). 

## 4. Discussion

UA and NLR, biomarkers of oxidative stress and inflammation, showed positive interactions to increase cardiac death risk in CAD, an effect that is greater than their summation. Instead, the risk of HE is essentially due to the NLR contribution, although an interactive effect may be appreciated also for this outcome, suggesting that patients presenting elevated levels of both biomarkers may have an increased risk.

The role of UA in cardiometabolic disease is still controversial and discussed. Although previously viewed as an inert metabolic end-product of purine metabolism, UA is now considered a potent antioxidant, which may play a protective role against oxidative stress [2,3]. However, different experimental and in vitro data indicate that this biomarker behaves as many other antioxidant molecules, which can shift from the physiological antioxidant role to pro-oxidizing effects according to their level and to microenvironment characteristics [2]. Accordingly, several clinical and epidemiological data by us and other researchers have demonstrated the relationship between hyperuricemia and cardiometabolic risk and disease, thereby overwhelming the beneficial actions of UA and suggesting UA as a potential pharmacological target (e.g., xanthine oxidase inhibitors) in cardiometabolic patients, especially in those with hyperuricemia [2,3,13,14,15,16,17].

It is well known that chronic inflammation plays a key role in the development of insulin resistance, type 2 diabetes, atherosclerosis, and cardiovascular disease [5,18]. NLR, easily calculated by a simple blood count analysis, is an inexpensive, easy to perform, and, as such, widely available inflammation-related parameter [19]. This biomarker has been evaluated in cardiometabolic diseases, resulting in a reliable tool to improve risk stratification and assess outcomes in patients with different cardiometabolic conditions [5,19,20]. This biomarker, as well as neutrophil count, has been found especially elevated in AMI, as confirmed by the present results [21,22]. UA also induces inflammation, and, consistent with our results, previous data reported a significant association between NLR and UA in CAD and a common relationship with cardiometabolic risk factors, making the combined assessment of UA and NLR a promising tool in risk-stratify patients with respect to arterial stiffness in CAD patients, and in risk-stratifying STEMI patients with respect to TIMI risk score [7,12,23,24,25,26,27,28,29]. Interestingly, previous data evidenced the correlation between UA and NLR and how NLR may be a determinant of inflammation and atherosclerosis in chronic kidney disease patients [30]. Moreover, a very recent study found an additive interaction between UA and NLR on ischemic stroke recurrence [31].

However, to date, no studies have explored the UA, NLR, and CAD relationship together, and the additive interaction of the two biomarkers in CAD. This is an innovative point and a major strength of this research. We observed that UA and NLR are reciprocally correlated and represent an independent determinant for each other. Moreover, the use of RERI > 0, AP > 0, and S > 1 indicate a biological interaction. In particular, among the index considered, RERI was the best tool to estimate the biological interaction using a proportional hazards model [32]. RERI results suggested an interaction between UA and NLR both for cardiac death and HE. Although NLR mostly contributes to the risk of adverse outcomes, this effect was evidenced despite the lack of significance of hyperuricemia in the multivariate analysis for HE. AP evidenced that 30% of cardiac deaths (unadjusted model) can be explained by the interaction between high UA and high NLR, suggesting that the burden of mortality in dual-exposed patients may be significantly reduced by improving one of the exposure variables. Interestingly, although hyperuricemia is not a significant determinant of HE in the univariate analysis, an additive interaction with high NLR was observed, suggesting that patients with elevation of both biomarker levels have a worse risk of prognosis anyways. This is important information, as additive interaction has long been recognized as relevant for stakeholders (e.g., physicians and policymakers), because it may be helpful to identify patients who will benefit most from an intervention but are poorly exploited in clinical practice [33].

The present study presents some limits. UA is known to be affected by alcohol intake, diuretics, and renal function, but information related to alcohol intake was not available in the database. The use of diuretics and creatinine was not available in all subjects; thus, these variables could not be evaluated in the multivariate analysis. Nonetheless, as expected, in the subgroups of subjects in which this data were available, there was a significant association between UA and creatinine (*r* = 0.2, *p* < 0.001), and UA levels were higher in patients taking when compared with those not taking diuretics (6.6 ± 1.8 vs. 5.8 ± 1.4 mg/dL, *p* < 0.001). Thus, these parameters merit evaluation in future studies to better assess their role in determining UA levels and the association of this biomarker with other cardiovascular risk factors. One limitation is that it is a retrospective and single-center study, and verifying this additive interaction requires application in more populations. Moreover, there are no shared cut-offs for UA and NLR. In particular, the cut-offs used for hyperuricemia (>6 mg/dL in women and 7 mg/dL in men) are mainly based on the the saturation point; however, some data suggest that the adverse effects of hyperuricemia on cardiometabolic conditions could occur even at lower levels [34,35,36]. At the same time, as currently there is no shared cut-off for NLR, the 75th percentile was chosen because it represents the level commonly used for stratification of this biomarker [37,38,39]

## 5. Conclusions

In conclusion, although it is clear that correlation does not mean causation, the reciprocal combination of UA and NLR could participate in the onset and development of cardiometabolic alterations showing an additive interaction. More prospective population-based studies are needed in the general population and in patients with cardiovascular disease to determine whether an intervention, such as lifestyle modifications or medications that reduce UA and/or inflammation, will decrease the cardiometabolic risk and improve the outcome.

## Figures and Tables

**Table 1:** Baseline characteristics of the study population.

Clinical Characteristics	no-CAD	CAD	AMI	*p*
Number	806	1545	361	-
Age (years)	65 ± 11	68 ± 10	66 ± 12	<0.001
Males	466 (58)	1220 (79)	274 (76)	<0.001
Hypertension	437 (54)	923 (60)	188 (52)	<0.01
Type 2 Diabetes	162 (20)	515 (33)	139 (38)	<0.001
Dyslipidemia	416 (52)	1224 (79)	289 (80)	<0.001
Smoking habit (current or past)	276 (32)	717 (46)	181 (50)	<0.001
Obesity (>30 kg/m^2^)	221 (27)	349 (23)	75 (21)	<0.05
EF (%)	53 ± 12	52 ± 11	46 ± 11	<0.001
Multi-vessel disease	-	876 (57)	204 (87)	ns
NLR	2.6 ± 3.4	2.6 ± 2.6	5.3 ± 4.3	<0.001
UA (mg/dL))	6 ± 1.7	6.1 ± 1.6	6.1 ± 1.5	ns

Data are presented as mean ± standard deviation or *n* (percent). ns: Non-significant difference; EF: ejection fraction; NLR: neutrophil lymphocyte ratio; UA: uric acid.

**Table 2:** Multiple regression between UA and different risk factors.

	Univariate Analysis			Multivariate Analysis		
Variable	Std-Coeff	*t*-Value	*p*	Std-Coeff	*t*-Value	*p*
Age (years)	0.08	4.0	<0.001	0.09	4.7	<0.001
Male gender	0.18	9.4	<0.001	0.18	9.3	<0.001
Hypertension	0.05	2.5	<0.05	0.05	2.8	<0.01
Type 2 Diabetes	0.11	5.9	<0.001	0.05	2.8	<0.01
Dyslipidemia	−0.25	−1.3	ns	-	-	-
Smoking habit (current or past)	0.03	1.5	ns	-	-	-
BMI	0.15	8.0	<0.001	0.15	7.7	<0.001
EF (%)	−0.24	−13.0	<0.001	−0.21	−11.1	<0.001
Multi-vessel disease	0.06	2.9	<0.01	−0.36	−1.8	ns
NLR	0.07	3.7	<0.001	0.04	2	<0.05

BMI: body mass index; EF: ejection fraction; NLR: neutrophil lymphocyte ratio; ns: Non-significant difference.

**Table 3:** Multiple regression between NLR and different risk factors.

	Univariate Analysis			Multivariate Analysis		
Variable	Std-Coeff	*t*-Value	*p*	Std-Coeff	*t*-Value	*p*
Age (years)	0.11	5.5	<0.001	0.07	3.3	<0.01
Male gender	−0.002	−0.11	ns	-	-	-
Hypertension	−0.05	−2.49	<0.05	−0.03	−1.7	ns
Type 2 Diabetes	0.1	5.0	<0.001	0.08	4	<0.001
Dyslipidemia	−0.6	−2.9	<0.01	−0.05	−2.6	<0.01
Smoking habit (current or past)	−0.01	−0.4	ns	-	-	-
BMI	−0.1	−5.3	<0.001	−0.1	−5.2	<0.001
EF (%)	−0.14	−7.4	<0.001	−0.08	−4.2	<0.001
Multi-vessel disease	0.08	4.2	<0.001	0.04	2.1	<0.05
UA (mg/dL))	0.07	3.7	<0.001	0.04	2.1	<0.05

BMI: body mass index; EF: ejection fraction; NLR: neutrophil lymphocyte ratio; ns: Non-significant difference.

**Table 4:** Cox predictive model for cardiac death and hard events.

	Univariate Analysis		Multivariate Analysis	
	HR (95% CI)	*p*	HR (95% CI)	*p*
Cardiac Death				
Age (>68 years, 50th percentile)	4.5 (2.7–7.5)	<0.001	3.5 (2.1–5.8)	<0.001
Male gender	1.0 (0.7–1.6)	ns	-	-
Hypertension	0.8 (0.6–1.2)	ns	-	-
Type 2 Diabetes	1.9 (1.3–2.8)	<0.01	1.6 (1.1–2.4)	<0.05
Dyslipidemia	0.4 (0.3–0.6)	<0.001	0.5 (0.3–0.7)	<0.001
Smoking habit	1.1 (0.8–1.7)	ns	-	-
Obesity (>30 kg/m^2^)	0.3 (0.2–0.6)	<0.01	0.3 (0.2–0.7)	<0.01
EF (<50%)	3.6 (2.4–5.4)	<0.001	2.6 (1.7–3.9)	<0.001
CAD	1.9 (1.1–3.1)	<0.05	1.7 (1.0–2.9)	<0.05
NLR (>3.2, 75th percentile)	2.9 (2.0–4.4)	<0.001	1.8 (1.2–2.7)	<0.01
UA (>6 mg/dL in females, >7 mg/dL in males)	1.7 (1.2–2.6)	<0.01	1.2 (0.9–1.9)	ns
HE				
Age (>68 years, 50th percentile)	2.7 (2.0–3.7)	<0.001	2.3 (1.7–3.2)	<0.001
Male gender	1.2 (0.9–1.7)	ns	-	-
Hypertension	1.0 (0.8–1.3)	ns	-	-
Type 2 Diabetes	1.4 (1.0–1.8)	<0.05	1.2 (0.9–1.5)	ns
Dyslipidemia	0.6 (0.5–0.8)	<0.001	0.7 (0.5–0.9)	<0.01
Smoking habit	1.1 (0.9–1.5)	ns	-	-
Obesity (>30 kg/m^2^)	0.5 (0.4–0.8)	<0.01	0.6 (0.4–0.9)	<0.01
EF (<50%)	<0.001	ns	2.1 (1.6–2.8)	<0.001
CAD	2.1 (1.4–2.9)	<0.001	1.9 (1.3–2.7)	<0.01
NLR (>3.2, 75th percentile)	2.5 (1.9–3.2)	<0.001	1.8 (1.4–2.4)	<0.001
UA (>6 mg/dL in females, >7 mg/dL in males)	1.1 (0.9–1.)	ns	-	-

CAD: coronary artery disease; CI: confidence interval; EF: ejection fraction; HE: hard events; HR: hazard ratio; NLR: neutrophil lymphocyte ratio; UA: uric acid; ns: Non-significant difference.

**Table 5:** Interactive effect of UA and NLR on cardiac death.

Parameter	Unadjusted HR (95% CI) *p*	Estimate Value (95% CI)	Adjusted HR * (95% CI) *p*	Estimate Value * (95% CI)
low UA low NLR	Reference		Reference	
high UA low NLR	1.6 (0.9–2.8) <0.1		1.4 (0.8–2.4) ns	
low UA high NLR	2.8 (1.7–4.7) <0.001		2.3 (1.3–3.8) <0.01	
high UA high NLR	4.7 (2.7–8.2) <0.001		3.3 (1.9–5.8) <0.001	
Additive Interaction				
RERI		1.4 (−1.0–3.7)		0.7 (−1.1–2.4)
AP		0.3 (−0.1–0.7)		0.2 (−0.3–0.7)
SI		1.6 (0.3–2.8)		1.4 (0.1–2.7)
Multiplicative Interaction				
ratio of HRs		1.1 (0.5–2.4)		1.1 (0.4–2.3)

* Model adjusted for age and sex. AP: attributable proportion; HR: hazard ratio; RERI: relative excess risk; SI: synergy index; ns: Non-significant difference.

**Table 6:** Interactive effect of UA and NLR on hard events.

Parameter	Unadjusted HR (95% CI) *p*	Estimate Value (95% CI)	Adjusted HR * (95% CI) *p*	Estimate Value * (95% CI)
low UA low NLR	Reference		Reference	
high UA low NLR	0.9 (0.6–1.3) ns		0.8 (0.5–1.2) ns	
low UA high NLR	2.1 (1.5–2.9) <0.001		1.8 (1.3–2.6) ≤0.001	
high UA high NLR	2.9 (2.0–4.2) <0.001		2.3 (1.6–3.4) <0.001	
**Additive Interaction**				
RERI		0.9 (−0.2–2.1)		0.7 (−0.2–1.6)
AP		0.3 (0.01–0.6)		0.3 (−0.04–0.6)
SI		2.0 (0.2–3.7)		2.1 (−0.4–4.7)
**Multiplicative Interaction**				
ratio of HRs		1.6 (0.9–2.8)		1.6 (0.9–2.8)

* Model adjusted for age and sex. AP: attributable proportion; HR: hazard ratio; RERI: relative excess risk; SI: synergy index; ns: Non-significant difference.

## Data Availability

The data presented in this study are available in the article.
